# Long term safety of ADHD medication in patients with schizophrenia spectrum disorders

**DOI:** 10.1038/s41380-025-03080-3

**Published:** 2025-07-01

**Authors:** Jurjen J. Luykx, Olivier Corbeil, Olli Kärkkäinen, Antti Tanskanen, Ellenor Mittendorfer-Rutz, Jari Tiihonen, Heidi Taipale

**Affiliations:** 1 Department of Psychiatry, Amsterdam University Medical Center, Amsterdam, The Netherlands; 2 GGZ inGeest Mental Health Care, Amsterdam, The Netherlands; 3 Amsterdam Neuroscience (Mood, Anxiety, Psychosis, Stress & Sleep program) and Amsterdam Public Health (Mental Health program) research institutes, Amsterdam, The Netherlands; 4 Department of Psychiatry and Neuropsychology, School for Mental Health and Neuroscience, Maastricht University Medical Center, Maastricht, The Netherlands; 5 Faculty of Pharmacy, Université Laval, Quebec, QC Canada; 6 Department of Pharmacy, Quebec Mental Health University Institute, Quebec, QC Canada; 7 School of Pharmacy, University of Eastern Finland, Kuopio, Finland; 8 Department of Forensic Psychiatry, University of Eastern Finland, Niuvanniemi Hospital, Kuopio, Finland; 9 Department of Clinical Neuroscience, Karolinska Institutet, Stockholm, Sweden; 10 Center for Psychiatry Research, Stockholm City Council, Stockholm, Sweden

**Keywords:** Schizophrenia, Prognostic markers

## Abstract

Attention-deficit hyperactivity disorder (ADHD) is frequently comorbid with schizophrenia spectrum disorders (SSDs) and is associated with poorer outcomes. Yet, its pharmacological treatment in patients with SSDs has been hampered by safety concerns. We therefore examined whether psychiatric, cardiovascular and other medical outcomes are associated with the use of ADHD medications in people with SSDs (*N* = 131,476). The main outcome was all-cause hospitalization/mortality. Secondary outcomes were hospitalization for psychosis, somatic hospitalization, and cardiovascular hospitalization. Adjusted hazard ratios (aHRs) were calculated for the association between the outcomes and the different exposure categories (compared with non-use of ADHD medication) using within-individual Cox regression analyses. Lisdexamphetamine was associated with a decreased risk of all-cause hospitalization/mortality (aHR = 0.89, 95%CI = 0.84–0.94) and methylphenidate with a slightly increased risk (aHR = 1.04 [1.01–1.08]), while for the other exposures the 95%CI of the HRs encompassed 1. Atomoxetine was associated with a reduced risk of hospitalization for psychosis (aHR = 0.87 [0.78–0.98]), lisdexamphetamine with a reduced risk of somatic hospitalizations (aHR = 0.70 [0.58–0.84]), and ADHD polytherapy with an increased risk of somatic hospitalizations (aHR = 1.37 [1.07–1.74]). No other statistically significant associations were found between the exposures and outcomes (including cardiovascular hospitalizations). Furthermore, increased all-cause hospitalization/mortality risks for methylphenidate were only found with doses ≥95 mgs/day (aHR 1.08 [1.03–1.14]) or during use periods of this agent without concomitant use of an antipsychotic (aHR = 1.06 [1.01–1.12]). Finally, for methylphenidate and lisdexamphetamine, we found evidence of U-shaped associations between doses used and risks of all-cause hospitalization/mortality and psychosis. In conclusion, we find that for people with SSDs, the use of ADHD medication (particularly lisdexamphetamine in all dosages and long-acting methylphenidate in low to medium doses) is safer than generally conceived. The benefits of its use for patients with SSD and comorbid ADHD should therefore be weighed against the risks in a shared decision-making process aimed at improving patients’ chances of recovery.

## Introduction

Schizophrenia spectrum disorders (SSDs) are associated with a significant disease burden, both in terms of disability and morbidity [1]. In addition, physical and psychiatric comorbidities are very common in people with SSD, including an up to two-fold increased risk of cardiovascular disease and of cardiovascular-related death compared to the general population [2]. Antipsychotic treatment has been linked to improved outcomes, including lower chances of psychiatric hospitalization [3], and increased survival [1, 4]. Despite this, few people experience symptomatic remission after a first episode of psychosis, and considerably fewer achieve recovery [5]. While part of the solution for these low remission and recovery chances may lie in discovering new and more effective antipsychotics and improving access to non-pharmacological approaches, appropriate management of comorbidities, both physical and psychiatric, is critical given their significant prevalence and detrimental impact on recovery [6].

High rates (ranging from 10–57%) of co-occurring attention-deficit/hyperactivity disorder (ADHD) have been reported in people with SSDs [7], but this comorbidity has received little attention despite potentially important negative consequences, including relatively poor functional outcomes [8]. Possibly contributing to this under-recognition is the difficulty in differentiating attentional and other cognitive deficits that may be due to ADHD from those associated with SSDs themselves. Indeed, many cognitive (e.g., attention, executive function, and learning) and behavioral (e.g., agitation and impulsivity) disturbances are common to ADHD and SSDs or may be exacerbated by antipsychotic treatment. Notably, there do not appear to be any specific deficits that clearly distinguish these two conditions [9]. Another important clinical dilemma concerns the treatment of these two conditions when they are comorbid. The first-line pharmacological treatment of ADHD, i.e., stimulants (amphetamines and methylphenidate), has long been avoided in people with psychotic disorders out of safety concerns, including a suspected increased risk of psychotic exacerbation [8].

Although recent evidence hints at associations between ADHD medication use and lower rates of all-cause and unnatural-cause mortality in general populations with ADHD [10], this has not been studied in SSDs, where clinicians may be concerned about misuse due to heightened vulnerability to substance use disorders and the associated risks of adverse events, including (accidental) poisoning and death [11]. Cardiovascular events have also been linked to long-term use of ADHD medication [12]. Thus, given the high incidence of somatic illness generally and cardiovascular disease specifically in people with SSDs [2], concerns about the safety of ADHD medication when used by these patients may play a bigger role in clinical practice than when used by patients without SSDs.

Regarding the risk of psychosis related to the use of ADHD medication, this stems mostly from case series or from studies conducted in patients without a history of psychosis [13, 14]. To our knowledge, only two cohort studies have so far examined the risk of psychosis associated with the use of ADHD medications in people with pre-existing psychotic disorders. A first study assessed the risk of psychotic events following treatment with methylphenidate in individuals aged 12–30 years, among whom 409 had a previous history of psychosis [15]. A second study examined the risk of psychosis after initiation of ADHD medication in 2219 individuals diagnosed with SSDs [16]. Only people receiving concomitant antipsychotic treatment at the time of ADHD medication initiation were included. While the results of these two studies were reassuring in that they suggested that the risk of hospitalization for psychosis was not increased following initiation of ADHD medication, they are based on relatively small study populations or confined to individuals with very good adherence to antipsychotic treatment. In addition, important safety outcomes of ADHD medication use, such as cardiovascular disease and mortality [11, 12], have to our knowledge not yet been evaluated in people with SSDs, a population at increased risk for both [1, 2]. Moreover, follow-up in these studies was limited to one year, a period too short to thoroughly dissect longer-term outcomes. Finally, the safety of lisdexamphetamine in particular, a relatively new agent with several advantages over other formulations of amphetamines, including a reduced risk of abuse [17], has not yet been specifically evaluated in patients with SSD.

In light of the aforementioned knowledge gaps regarding the psychiatric and general medical safety of ADHD medication in people with a history of psychosis, we set out to examine whether psychiatric, cardiovascular and other medical outcomes are associated with the use of ADHD medications in people with SSDs. Thus, we comprehensively dissected psychiatric and somatic risks during use periods of ADHD medications across a range of clinical scenarios, e.g., when prescribed in specific dosages and when used without the concomitant use of antipsychotics.

## Methods

### Datasets and ethics

Reporting of this study conforms to the Strengthening the Reporting of Observational Studies in Epidemiology (STROBE) statement [18]. We conducted a population-based cohort study, employing data from several Swedish health and population registers. Using a pseudonymized personal identification number, data were linked from the National Patient Register (NPR; including data on inpatient stays, specialized outpatient care visits, and diagnoses); Microdata for Analyses of Social Insurance (MiDAS) register (including data on sickness absence, disability pension, and associated diagnoses); and the Prescribed Drug Register (PDR), consisting of data on all dispensed prescription drugs since July 2005. The project was approved by the Regional Ethical Review Board, Karolinska Institutet, Stockholm, Sweden (Dnr: 2007/762-31 and Dnr 2021-06441-02). We abided by the declaration of Helsinki.

### Study population

The study cohort was retrieved from the aforementioned NPR and MiDAS registers. It included all individuals aged 16–65 years diagnosed with a SSD according to the International Classification of Diseases, version 10 [ICD-10] in Sweden from 2006 until 2021, i.e. F20 (schizophrenia), F21 (schizotypal disorder), F22 (delusional disorders), F23 (brief psychotic disorder), F24 (shared psychotic disorder), F25 (schizoaffective disorder), and F29 (unspecified psychosis). All these disorders match the DSM-5 SSDs, with the exception of the catatonic disorders, that are not included in ICD SSDs but are currently listed under SSDs in the DSM-5. Cohort entry was defined as the date of first diagnosis for those diagnosed during this time interval, and January 1, 2006 for those diagnosed before. The follow-up ended at death, emigration or end of data linkage (December 31, 2021), whichever occurred first.

### Exposures

The exposures were use of ADHD medications, defined as WHO’s Anatomical Therapeutic Chemical (ATC) classification code N06BA, including methylphenidate, amphetamine, dexamphetamine, lisdexamphetamine, atomoxetine, and modafinil. Drug use periods, i.e., estimates of when drug use started and ended, were formed with the PRE2DUP method that has been described in detail before [19]. In brief, after modelling each ATC code, we created time periods when two or more ADHD medications were used and coded these as ADHD polytherapy, while specific medications analyzed are referred to as ADHD monotherapy. Different exposure categories were then formed: (1) ADHD medication as a group (i.e., use of ≥1 of the following, in any possible combination: atomoxetine, modafinil, methylphenidate, amphetamine, dexamphetamine, and lisdexamphetamine); (2) stimulants only (i.e., use of ≥1 of the following, in any possible combination: methylphenidate, amphetamine, dexamphetamine, and lisdexamphetamine); (3) specific ADHD medications (i.e., methylphenidate, amphetamine, dexamphetamine, lisdexamphetamine, atomoxetine, and modafinil in monotherapy); (4) ADHD medication polytherapy (i.e., a combination of ≥2 of any of the agents listed under 1).

### Outcomes

The main outcome was all-cause hospitalization/mortality. Secondary outcomes were hospitalization for psychosis (ICD-10 codes F20–F29), somatic hospitalization (A00-N99, excluding F00–F99), and cardiovascular hospitalization (I00–I99). Data on hospitalizations were extracted from the NPR, defined as an inpatient stay of at least 24 h.

### Statistical analysis

Analyses were conducted using a within-individual design, where all time-invariant factors are automatically controlled for by the design [3]. ADHD medication use periods were thus compared with non-use periods of ADHD medication within the same individual, which minimizes the risk of selection bias. Analyses were adjusted for time-dependent covariates: time since cohort entry, temporal order of the ADHD medications used, and concomitant use of psychotropic drugs, namely antipsychotics (ATC N05A excluding lithium N05AN01), antidepressants (N06A), benzodiazepines and related drugs (N05BA, N05CD, N05CF), mood stabilizers (carbamazepine N03AF01, valproic acid N03AG01, lamotrigine N03AX09 and lithium N05AN01), and drugs for addictive disorders (N07BB, N07BC). In these within-individual analyses, outcomes were treated as recurring events and analyzed with stratified Cox regression models [20].

To corroborate the robustness of the findings, we first investigated associations between medication use and risk of all-cause hospitalization/mortality in traditional between-individual Cox models. Since within-individual analyses only directly apply to the subset of individuals from the study cohort who used ADHD medication, between-individual analyses were also performed in the whole study cohort, to corroborate whether applying this method results in similar findings as the within-individual analyses. In addition to the aforementioned time-dependent covariates, these between-individual analyses were adjusted for age, sex, disability pension, number of previous hospitalizations for psychosis, diagnosis of ADHD, substance use disorder, previous suicide attempts, and previous use of clozapine. To examine whether antipsychotic use during ADHD medication use attenuates risks for the main outcome, we compared the risk of this outcome during antipsychotic use (i.e., any ≥1 antipsychotic) with non-antipsychotic use periods, using periods of no antipsychotic and no ADHD medication use as the reference.

For the secondary outcome of somatic hospitalization, an additional analysis using a similar within-individual design was performed specifically for hospitalization for neurological conditions (ICD-10 codes G00-G99), given conflicting evidence about a potential association between ADHD medication use and increased risk of neurological adverse events, including a lowered seizure threshold [21–23].

To deepen the understanding of a possible dose-response relationship between the two most commonly used ADHD medications and the two most frequent outcomes, using a within-individual design we examined possible associations between methylphenidate and lisdexamphetamine and all-cause hospitalization/mortality on the one hand, and hospitalization for psychosis on the other, stratified by dose categories (i.e., <45, 45–<75, 75–<95, and ≥95 mg/day). Then, to examine possible associations with medications’ onset of action, short- (i.e., immediate release) and long-acting (i.e., sustained/extended release) formulations of methylphenidate were modelled separately as short-acting only, long-acting only, and a combination of the two. These analyses were also additionally stratified using the previously described dose categories. For dexamphetamine we had too few observations to allow for such an analysis.

Finally, to minimize the chances of possible carryover effects of medication on the outcome impacting our findings, we assessed the occurrence of the primary outcome >30 days after discontinuation of ADHD medication.

The results are reported as adjusted hazard ratios (aHRs) and corresponding 95% CIs and p-values. Data management and analyses were conducted with SAS 9.4.

## Results

### Study population characteristics

The study cohort included 131,476 individuals with SSDs, 9416 of whom used ADHD medications during a mean follow-up period of 8.9 years (standard deviation [SD] 5.1) and for 25,601 person-years of use. The mean age of these ADHD medication users with SSD was 31.7 years (SD 10.8), and 37.8% (*N* = 3558) were women (Table 1). Slightly less than half (45.6%) had initiated ADHD medication either prior to cohort entry or in the following year. The most common ADHD medications used were methylphenidate (67.2%; *N* = 6326), lisdexamphetamine (38.1%; *N* = 3587), and atomoxetine (24.5%; *N* = 2312). Approximately one third (*N* = 3074) of all ADHD medication users had an ADHD diagnosis at cohort entry, while this proportion increased to 87.3% (*N* = 8219) at the end of the follow-up period.

**Table 1:** Characteristics of ADHD medication users among persons with SSDs (*N* = 9416).

	% (N)
Age at cohort entry, mean (SD)	31.7 (10.8)^a^
Female sex	37.8 (3558)
Type of SSD diagnosis	
Schizophrenia (F20)	16.1 (1520)
Acute and transient psychosis (F23)	27.4 (2580)
Unspecified psychosis (F29)	35.9 (3382)
Other (F21, F22, F24, F25)	20.5 (1934)
Sickness absence during the year preceding cohort entry	
0 days	81.3 (7656)
1–90 days	9.0 (847)
>90 days	9.7 (913)
Disability pension at cohort entry	35.8 (3368)
Time to ADHD medication initiation since cohort entry	
0 year	28.3 (2661)
>0–1 year	17.4 (1637)
>1–5 years	31.8 (2991)
≥5 years	22.6 (2127)
ADHD diagnosis^b^	87.3 (8219)
Any substance use disorder^b^	63.1 (5945)

*ADHD* attention-deficit/hyperactivity disorder, *SD* standard deviation, *SSD* schizophrenia spectrum disorder.

^a^Age in years is reported here.

^b^These diagnoses were made by the treating physician at some point during follow-up.

### Within-individual analyses: risk of all-cause hospitalization and mortality

**Fig. 1:**
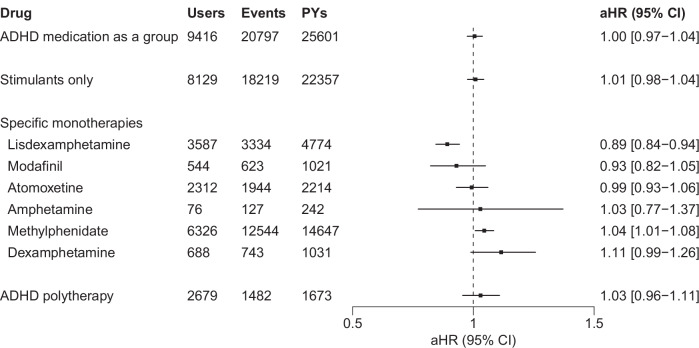
Risks of all-cause hospitalization/mortality associated with the use of ADHD medications compared with non-use of ADHD medications among persons with SSDs (*N* = 9416), with monotherapy ADHD medications, ordered by increasing risks. ADHD attention-deficit/hyperactivity disorder, aHR adjusted hazard ratio, CI confidence interval, SSDs schizophrenia spectrum disorders, PYs person-years, aHR hazard ratios adjusted for time-dependent covariates (i.e., time since cohort entry, temporal order of the ADHD medications used, and use of concomitant psychotropic drugs) in within-individual analyses.

There were a total of 20,797 all-cause hospitalization/mortality events among ADHD medication users over the follow-up period, of which 838 were deaths (Fig. 1). The most common causes of death were suicides and accidental poisonings (covering 54.0% of deaths).

The 95% CIs of the aHRs for the use of ADHD medication as a group, stimulants only, and ADHD polytherapy all encompassed 1. For specific medications in monotherapy, the use of lisdexamphetamine was associated with a reduced risk of all-cause hospitalization/mortality (aHR 0.89, 95% CI 0.84–0.94) and the use of methylphenidate with a slightly increased risk (aHR 1.04, 95% CI 1.01–1.08); the 95% CIs of the aHRs for other monotherapies encompassed 1.

When compared to periods of non-use of both antipsychotic treatment and ADHD medications, the use of lisdexamphetamine without an antipsychotic remained associated with a reduced risk of all-cause hospitalization/mortality (aHR 0.89, 95% CI 0.82–0.96). This hazard ratio was also similar to the observation for the combined use of lisdexamphetamine and an antipsychotic (aHR 0.87, 95% CI 0.82–0.94; Supplementary Table 1). In contrast, methylphenidate used without an antipsychotic remained associated with an increased risk of all-cause hospitalization/mortality (aHR 1.06, 95% CI 1.01–1.12), but not when used concomitantly with an antipsychotic (aHR 1.01, 95% CI 0.97–1.06). For all other specific ADHD medications and ADHD polytherapy, the 95% CIs of the aHRs during antipsychotic use and non-use encompassed 1, suggesting that overall risks for ADHD medications were similar for use periods with and without an antipsychotic, except for methylphenidate use.

### Within-individual analyses: risks of hospitalization for psychosis and for somatic conditions

**Fig. 2:**
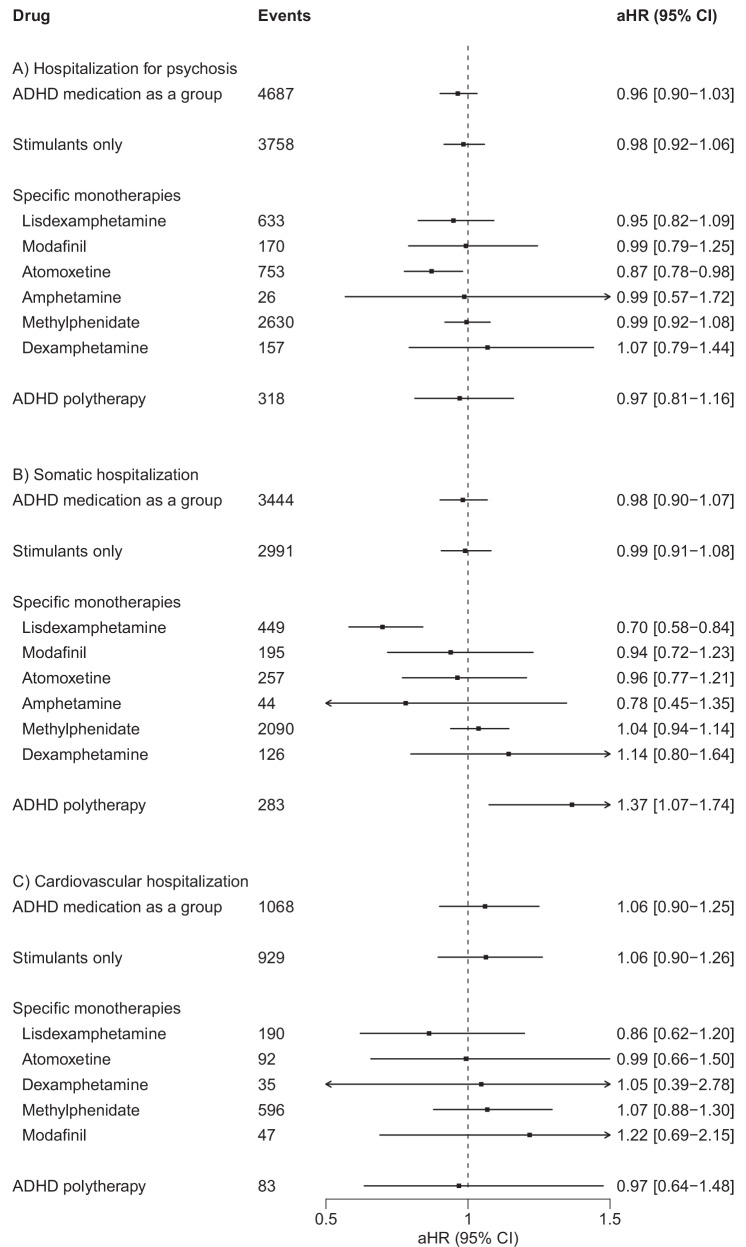
Associations between the use of ADHD medications among persons with SSDs (*N* = 9416), ordered in accordance with Fig. 1. **A** Risk of hospitalization for psychosis; **B** risk of somatic hospitalization; **C** risk of cardiovascular hospitalization. ADHD attention-deficit/hyperactivity disorder, aHR adjusted hazard ratio, CI confidence interval, SSDs schizophrenia spectrum disorders, aHR hazard ratios adjusted for time-dependent covariates (i.e., time since cohort entry, temporal order of the ADHD medications used, and use of concomitant psychotropic drugs) in within-individual analyses.

We observed 4687 hospitalizations for psychosis occurring during periods of ADHD medication use. The use of atomoxetine was associated with a reduced risk of hospitalization for psychosis (aHR 0.87, 95% CI 0.78–0.98), whereas for the remaining exposures the 95% CIs all encompassed 1 (Fig. 2A).

The most common causes of somatic hospitalization were: cutaneous abscess, furuncle and carbuncle (ICD-10 code L02; *N* = 347), epilepsy and recurrent seizures (ICD-10 code G40; *N* = 320), and pneumonia, unspecified organism (ICD-10 code J18; *N* = 305; Supplementary Table 2). For the outcome of somatic hospitalizations, lisdexamphetamine use was associated with a decreased risk (aHR 0.70, 95% CI 0.58–0.84) and ADHD polytherapy with an increased risk (aHR 1.37, 95% CI 1.07–1.74; Fig. 2B). For hospitalizations for neurological conditions specifically, the 95% CIs of the aHRs for all exposures included 1 (Supplementary Fig. 1). We observed the same pattern of wide confidence intervals for cardiovascular hospitalizations (Fig. 2C).

### Between-individual analyses: risks of all-cause hospitalization and mortality

Compared with non-use of ADHD medications, the risk of all-cause hospitalization/mortality in between-individual analyses performed among ADHD medication users only (*N* = 9416) was reduced with the use of lisdexamphetamine (aHR 0.82, 95% CI 0.78–0.87; Supplementary Fig. 2). For the remaining exposures, the 95% CIs of the aHRs all encompassed 1.

**Fig. 3:**
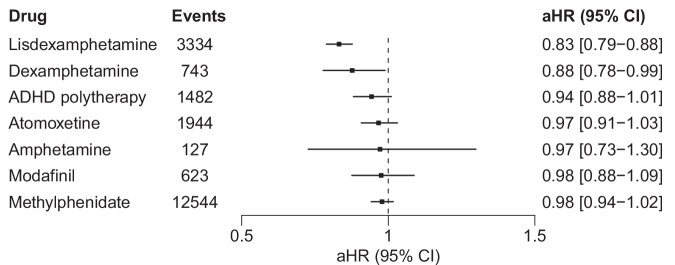
Risk of all-cause hospitalization/mortality associated with the use of specific ADHD medications among persons with SSDs in the whole study cohort (*N* = 131,476), using between-individual analyses. ADHD attention-deficit/hyperactivity disorder, aHR adjusted hazard ratio, CI confidence interval, SSDs schizophrenia spectrum disorders, aHR hazard ratios adjusted for age, sex, disability pension, number of previous hospitalizations for psychosis, diagnosis of ADHD, substance use disorder, previous suicide attempts, previous use of clozapine, time-varying use of antipsychotics, antidepressants, mood stabilizers, drugs for addictive disorders, benzodiazepines and related drugs, and temporal order of ADHD drugs used in between-individual analyses.

In between-individual analyses performed in the whole study cohort (*N* = 131,476), the use of lisdexamphetamine (aHR 0.83, 95% CI 0.79–0.88) and dexamphetamine (aHR 0.88, 95% CI 0.78–0.99) were associated with decreased risks of all-cause hospitalization/mortality (Fig. 3). The 95% CIs of the aHRs for all other exposures included 1.

### Dose-response analyses

**Fig. 4:**
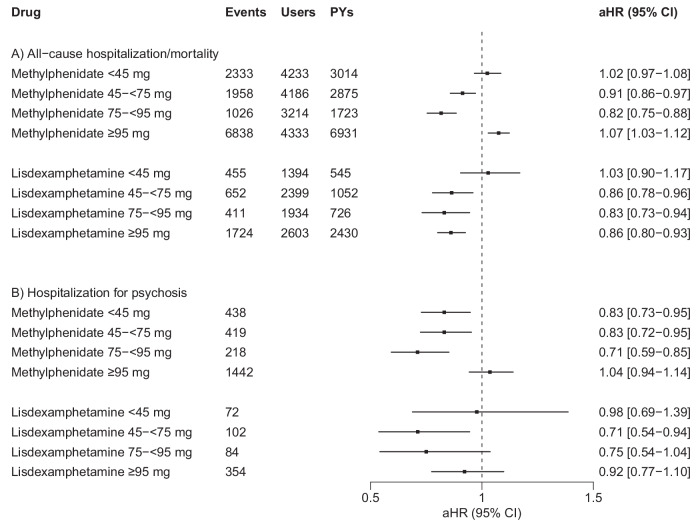
Associations between the use of methylphenidate and lisdexamphetamine, stratified by dose categories among persons with SSDs (*N* = 9416). **A** Risk of all-cause hospitalization/mortality; **B** risk of hospitalization for psychosis. aHR adjusted hazard ratio, CI confidence interval, SSDs schizophrenia spectrum disorders, PYs person-years, aHR hazard ratios adjusted for time-dependent covariates (i.e., time since cohort entry, temporal order of the ADHD medications used, and use of concomitant psychotropic drugs) in within-individual analyses. Dose categories are expressed in mg/day.

When examining possible associations between different dose categories and all-cause hospitalization/mortality, the 95% CIs of the aHRs for doses <45 mg/day encompassed 1 for both methylphenidate and lisdexamphetamine (Fig. 4A). The use of methylphenidate at doses between 45–<75 mg/day (aHR 0.91, 95% CI 0.86–0.97) and 75–<95 mg/day (aHR 0.82, 95% CI 0.75–0.88), as well as the use of lisdexamphetamine at doses between 45–<75 mg/day (aHR 0.86, 95% CI 0.78–0.96), 75–<95 mg/day (aHR 0.83, 95% CI 0.73–0.94), and ≥95 mg/day (aHR 0.86, 95% CI 0.80–0.93) were associated with reduced risks of all-cause hospitalization/mortality (Fig. 4A). The use of methylphenidate at doses ≥95 mg/day was associated with increased risk of all-cause hospitalization/mortality (aHR 1.07 [1.03–1.12]; Fig. 4A).

For hospitalization for psychosis, the use of methylphenidate at doses <45 mg/day (aHR 0.83, 95% CI 0.73–0.95), 45–<75 mg/day (aHR 0.83, 95% CI 0.72–0.95), and 75–<95 mg/day (aHR 0.71, 95% CI 0.59–0.85), as well as the use of lisdexamphetamine at doses between 45–<75 mg/day (aHR 0.71, 95% CI 0.54–0.94) were associated with reduced risks (Fig. 4B). For other dose categories, the 95% CIs of the aHRs included 1 (Fig. 4B).

### Short-acting and long-acting formulations of methylphenidate

**Fig. 5:**
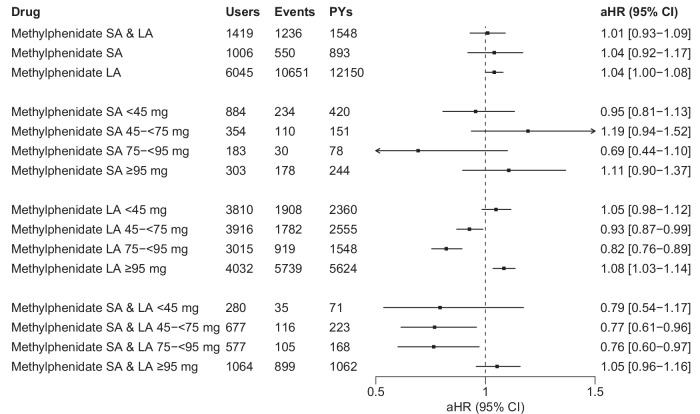
Risks of all-cause hospitalization/mortality associated with the use of long-acting (LA) vs. short-acting (SA) forms of methylphenidate, and stratified by dose categories, among persons with SSDs (*N* = 9416). Methylphenidate SA & LA = use periods when short-acting and long-acting methylphenidate were used concomitantly; aHR adjusted hazard ratio, CI confidence interval, LA long-acting, SSDs schizophrenia spectrum disorders, PYs person-years, SA short-acting, aHR hazard ratios adjusted for time-dependent covariates (i.e., time since cohort entry, temporal order of the ADHD medications used, and use of concomitant psychotropic drugs) in within-individual analyses.

In within-individual analyses among ADHD medication users, the 95% CIs of the aHRs for the use of short-acting only, of long-acting only, or of both formulations of methylphenidate and the risk of all-cause hospitalization/mortality all encompassed 1 (Fig. 5). When examining different dose categories, the use of long-acting formulations of methylphenidate at doses between 45–<75 mg/day (aHR 0.93, 95% CI 0.87–0.99) and 75–<95 mg/day (aHR 0.82, 95% CI 0.76–0.89) were associated with reduced risks, and doses ≥95 mg/day (aHR 1.08, 95% CI 1.03–1.14) with an increased risk (Fig. 5). For the concomitant use of both formulations of methylphenidate, doses between 45–<75 mg/day (aHR 0.77, 95% CI 0.61–0.96) and 75–<95 mg/day (aHR 0.76, 95% CI 0.60–0.97) were associated with reduced risks (Fig. 5). For other dose categories, the 95% CIs of the aHRs included 1 (Fig. 5).

### Accounting for possible carryover effects of ADHD medication use on main outcome

When omitting the first 30 days of ADHD medication non-use after their discontinuation from within-individual analyses in ADHD medication users, use periods of lisdexamphetamine (aHR 0.74, 95% CI 0.70–0.79), modafinil (aHR 0.77, 95% CI 0.68–0.88), atomoxetine (aHR 0.78, 95% CI 0.73–0.84), methylphenidate (aHR 0.91, 95% CI 0.87–0.94), and ADHD polytherapy (aHR 0.76, 95% CI 0.70–0.82) were all associated with reduced risks of all-cause hospitalization/mortality (Supplementary Fig. 3). For the use of amphetamine and dexamphetamine, the 95% CIs of the aHRs encompassed 1.

## Discussion

Here, by analyzing an unprecedented number of individuals with SSDs who used ADHD medication over a mean follow-up period of almost 9 years, we found evidence supporting the long-term safety of these medications with regards to several psychiatric and general medical outcomes. While the use of methylphenidate (all doses combined) and ADHD medication polytherapy were associated with slightly increased risks of all-cause hospitalization/mortality and somatic hospitalization, respectively, lisdexamphetamine was associated with lower risks for both outcomes relative to non-use. For all other ADHD medications examined, we found no increased risks of adverse outcomes, including for hospitalization due to neurological and cardiovascular conditions. We found no evidence of increased risk of all-cause hospitalization/mortality for all of the ADHD medications when used concomitantly with antipsychotics. With regards to dose, we found evidence of a U-shaped dose-response relationship for lisdexamphetamine, with dose categories 45–<75 and 75–<95 mg/day consistently showing the strongest negative associations with adverse outcomes. For methylphenidate, a similar pattern was observed, but in contrast to the observations for lisdexamphetamine, the ≥95 mg/day category was associated with an increased risk of all-cause hospitalization/mortality. Finally, when examining short and long-acting formulations of methylphenidate, the long-acting forms used at ≥95 mg daily were associated with increased risks of all-cause hospitalization/mortality, while the two middle long-acting dose categories were associated with lower such risks.

Although there is a paucity of data on the long-term safety of ADHD medication use among individuals with SSDs, our results are consistent with findings in other populations. This includes reduced risks of all-cause mortality and hospitalizations due to unintentional injuries associated with ADHD medication use found in individuals aged ≤ 24 years with ADHD [24]. Among Swedish residents with ADHD aged 16–65 years, dexamphetamine, lisdexamphetamine, methylphenidate, and ADHD polytherapy were associated with a decreased risk of suicide attempt and death [25]. Similarly, the use of methylphenidate and lisdexamphetamine were both associated with reduced risks of hospitalization or death due to any cause among Swedish residents aged 16–65 years with amphetamine use disorder [26]. In our study, the first to examine these outcomes in SSDs, only lisdexamphetamine use was associated with a reduced risk of both all-cause hospitalization/mortality and somatic hospitalizations. Of note, lisdexamphetamine is a prodrug of dextroamphetamine with a delayed onset of action even when administered intravenously, which has been suggested to reduce its potential for misuse [17]. Therefore, the reduced risk of all-cause hospitalization/mortality and somatic hospitalization with lisdexamphetamine use could be partly due to a more limited parenteral abuse potential, in turn leading to less hospitalizations/mortality due to substance abuse and accidental poisonings. However, it is plausible that other mediating factors may also be involved in the association, including the potential superiority of lisdexamphetamine and amphetamines over other pharmacologic options in the treatment of ADHD symptoms [27], and the possibility that lisdexamphetamine is effective in the treatment of core schizophrenia symptoms [28], as well as the superiority of amphetamines over methylphenidate in reducing relapse risks in schizophrenia [14]. In addition, since methylphenidate is recommended as first-line treatment for ADHD in Sweden and lisdexamphetamine as second-line treatment [29], it is not likely that people receiving lisdexamphetamine prescriptions are at lower risks of adverse safety outcomes than the rest of our study population. In addition, the analyses were adjusted for the temporal order in which ADHD medications were used to account for such potential differences.

More specifically in people with SSDs, the use of ADHD medication has been linked to concerns about the risk of psychotic exacerbation [8]. Our findings of no increased risk of hospitalization for psychosis associated with ADHD medication use in this study are consistent with previous evidence from observational studies [13]. As previously mentioned, no increased risk of psychotic events was found during the 12 weeks following methylphenidate initiation compared with the 12 weeks before initiation, albeit in a very small sample of adolescents and young adults with a history of psychosis [15]. Moreover, results from a cohort study using Quebec (Canada) registries suggested a reduced risk of hospitalization for psychosis associated with ADHD medication use in the year following initiation in individuals with SSDs receiving concomitant antipsychotic treatment [16]. Although these results provide reassuring evidence on the safety of ADHD medication use in terms of psychotic relapse, potential beneficial effects on this outcome were not found in our study and thus their use should remain limited to patients expected to benefit the most from it, such as those with comorbid ADHD (and possibly in the future, when evidence accrues, in those with cognitive impairment). Our observations also support the hypothesis that the co-administration of ADHD medication and antipsychotic treatment may not necessarily be antagonistic; instead, ADHD medication may help remediate downregulated tonic levels of dopamine in the prefrontal cortex (pro-cognitive effect), with antipsychotic treatment providing sufficient D2 antagonist activity in the associative striatum to prevent the exacerbation of positive psychotic symptoms [8, 30].

Finally, the lack of evidence suggesting an increased risk of cardiovascular hospitalization with the use of ADHD medication reported here contrasts with the results of a recent case-control study. In the latter study, which included 278,027 Swedish residents aged 6–64 years with incident ADHD or initiation of ADHD medication, long-term cumulative exposure to these medications (>1 year) was associated with increased odds of developing any cardiovascular disease over a median follow-up period of 4.1 years [12]. On the one hand, these are two different outcomes and distinct populations from ours, which may partly explain these contrasting results. On the other hand, our results are consistent with a previous meta-analysis of 19 observational studies with over 3.9 million participants, finding no association between ADHD medication use and the risk of cardiovascular disease across all age groups [31], although not specifically in individuals with a history of psychosis. Whether our findings reflect the absence of a causal relationship between ADHD medications and cardiovascular disease, a low severity of cardiovascular conditions not necessitating admission, or SSD-specific epidemiological aspects [1, 2] remains to be further investigated.

Strengths of this study include access to extensive and complementary Swedish nationwide registries, allowing identification of a significant study cohort size with long-term follow-up and extensive information on clinically relevant outcomes, including mortality. In contrast to randomized clinical trials, where it is not readily feasible to study rare outcomes that occur only after long-term exposure, observational studies such as this one allow for the inclusion of individuals who are typically underrepresented in clinical trials [32]. Although observational studies are prone to bias, particularly selection and confounding, within-individual analyses mitigate both by comparing periods of drug use and periods of non-use in the same individual. We also conducted traditional between-individual analyses adjusted for key potential confounders, whose results turned out to be consistent with those of the main analyses. Potential carryover bias was also examined in an additional analysis, which suggests that the main findings were not caused by an overrepresentation of events occurring in the 30 days following ADHD medication non-use periods, further strengthening the robustness of the results. Nonetheless, some limitations may be borne in mind when interpreting the results of this study. As briefly mentioned above, due to the nature of the methods the results reported here provide no evidence of causal effects. For example, it cannot be ruled out that the reason why some individuals did not use ADHD medications during follow-up was that they had previously experienced adverse drug reactions. In addition, it was not possible to perform statistically relevant subgroup analyses based on the type of SSD, mainly because of the relatively small number of individuals with a formal diagnosis of schizophrenia. On the one hand, grouping all SSDs allowed for increased statistical power and offered the advantage of increased clinical generalizability to the general group of patients with psychotic disorders, but came at the cost of limited specificity of findings to specific psychotic disorders. On the other hand, our approach of grouping all SSDs also makes sense diagnostically; while diagnostic stability of SSDs as a group is generally high [33], variations of specific diagnoses within this group are common over time [34]. Furthermore, too few seizure events were present in our dataset to conduct analyses for the outcome of seizures specifically. Finally, not all ADHD medication users in this study had a diagnosis of ADHD, although this may be an underestimate due to diagnoses made in private practice that are unavailable to public health registries. It is therefore not possible to confirm whether the off-label vs. on-label use of ADHD medication has any impact on the long-term safety of ADHD medication in people with SSDs.

Our results set the stage for future research in several ways. There is currently little to no guidance on the optimal treatment of comorbid ADHD in people with SSDs: while existing ADHD treatment guidelines recognize the need of addressing first the most severe disorder, for instance SSDs, they generally do not provide guidance regarding pharmacological treatment other than recognizing the risk of psychosis associated with ADHD medication [35]. Our findings provide evidence that ADHD medication use in individuals with SSDs may be safer than generally conceived, which in turn may inform treatment guidelines as well as decision-making in real-world settings. Questions to be addressed in future research relate to the identification of potential factors predictive of either adverse or favorable outcomes among people with SSDs. This includes collecting large samples to examine whether specific antipsychotics and adherence may improve the safety and efficacy of ADHD medication use in people with SSDs. In addition, randomized trials may determine whether lisdexamphetamine indeed outperforms methylphenidate in terms of effectiveness and tolerability. Furthermore, our findings suggest that for methylphenidate, the use of long-acting should be preferred over short-acting formulations and the safest doses may be in the range of 45–<95 mg/day, with an increased risk of all-cause events above that range. For lisdexamphetamine use, the most beneficial doses were above 45 mg/day, with beneficial outcomes for all-cause hospitalization/mortality maintained beyond 95 mg/day, and no evidence of an increased risk of hospitalization for psychosis even at these higher doses. Randomized studies may examine dose-response relationships further, if statistical power allows. Finally, by including neurocognition as an outcome, randomized clinical trials may dissect the possible cognitive benefits of ADHD medication in SSDs.

In conclusion, we comprehensively dissected associations between the use of ADHD medication and a range of safety outcomes in patients with SDDs. The main clinical implication of our findings is that for people with SSDs, the use of ADHD medication, particularly lisdexamphetamine and long-acting methylphenidate at low to medium doses, is safer than generally conceived. Moreover, although our methods do not allow for causal inference, based on our findings we advise against the use of methylphenidate generally in patients with SSDs not using an antipsychotic, and against the use of high-dose (≥95 mg daily) methylphenidate specifically in all patients with SSDs. The benefits of effective treatment of comorbid ADHD should be weighed against the risks in a personalized, shared decision-making process aimed at improving patients’ chances of recovery.

## Supplementary information

Supplementary material

## Data Availability

The project utilized data from the REWHARD consortium, supported by the Swedish Research Council (VR grant number. 2017-00624). These data cannot be made publicly available due to privacy regulations. According to the General Data Protection Regulation, the Swedish law SFS 2018:218, the Swedish Data Protection Act, the Swedish Ethical Review Act, and the Public Access to Information and Secrecy Act, these types of sensitive data can only be made available for specific purposes, including research, that meet the criteria for access to these type of sensitive and confidential data as determined by a legal review. Readers may contact Professor Kristina Alexanderson (kristina.alexanderson@ki.se) regarding the data.

## Code availability

Code used for statistical analysis is available upon request to the corresponding author.
