# Supplementary material for: Long term safety of ADHD medication in patients with schizophrenia spectrum disorders
Source: Mol Psychiatry. 2025 Jul 1;30(10):4859–67. doi: 10.1038/s41380-025-03080-3 (PMC12436181; doi:10.1038/s41380-025-03080-3)
Supplement: Supplementary file 1 — Supplementary material [file 41380_2025_3080_MOESM1_ESM.docx]

Supplementary material

**Long term safety of ADHD medication in patients with**

**schizophrenia spectrum disorders**

Jurjen J. Luykx^1-4*^, Olivier Corbeil^5,6*^, Olli Kärkkäinen^7^, Antti Tanskanen^8-10^, Ellenor Mittendorfer-Rutz^9^, Jari Tiihonen^8-10^, Heidi Taipale^7-10^

1. Department of Psychiatry, Amsterdam University Medical Center, Amsterdam, the Netherlands.
2. GGZ inGeest Mental Health Care, Amsterdam, The Netherlands.
3. Amsterdam Neuroscience (Mood, Anxiety, Psychosis, Stress & Sleep program) and Amsterdam Public Health (Mental Health program) research institutes, Amsterdam, the Netherlands.
4. Department of Psychiatry and Neuropsychology, School for Mental Health and Neuroscience, Maastricht University Medical Center, Maastricht, the Netherlands.
5. Faculty of Pharmacy, Université Laval, Quebec, Canada.
6. Department of Pharmacy, Quebec Mental Health University Institute, Quebec, Canada.
7. School of Pharmacy, University of Eastern Finland, Kuopio, Finland.
8. Department of Forensic Psychiatry, University of Eastern Finland, Niuvanniemi Hospital, Kuopio, Finland.
9. Department of Clinical Neuroscience, Karolinska Institutet, Stockholm, Sweden.
10. Center for Psychiatry Research, Stockholm City Council, Stockholm, Sweden.

*shared first authors

**Corresponding authors:**

Drs. Heidi Taipale at [heidi.taipale@uef.fi](mailto:heidi.taipale@uef.fi) and Jurjen Luykx at [j.j.luykx@amsterdamumc.nl](mailto:j.j.luykx@amsterdamumc.nl)

Contents

[**Supplementary Table 1.** *Risk of all-cause hospitalization/mortality associated with the use of specific ADHD medications with and without concomitant antipsychotic treatment among persons with SSD (N=9,416) using between-individual analyses.* 3](#_Toc182558132)

[**Supplementary Table 2.** *Most common causes of somatic hospitalization at 3 characters level among persons with SSD during periods of ADHD medication use.* 4](#_Toc182558133)

[**Supplementary Figure 1.** *Risk of hospitalization for neurological conditions* *associated with the use of ADHD medications among persons with SSDs (N=9,416).* 5](#_Toc182558134)

[**Supplementary Figure 2.** *Risk of all-cause hospitalization/mortality associated with the use of specific ADHD medications among persons with SSD (N=9,416) using between-individual analyses.* 6](#_Toc182558135)

[**Supplementary Figure 3.** *Risk of all-cause hospitalization/mortality associated with the use of specific ADHD medications among persons with SSDs (N=9,416), removing the first 30 days of ADHD medications non-use following their discontinuation.* 7](#_Toc182558136)

#

# Supplementary Table 1. *Risk of all-cause hospitalization/mortality associated with the use of specific ADHD medications with and without concomitant antipsychotic treatment among persons with SSD (N=9,416) using between-individual analyses.*

|  | **Without antipsychotic** | | | | **With antipsychotic** | | | |
| --- | --- | --- | --- | --- | --- | --- | --- | --- |
|  | Events | Users | Person-years | *aHR (95% CI) | Events | Users | Person-years | *aHR (95% CI) |
| Amphetamine | 68 | 57 | 168 | 0.84 (0.55-1.30) | 59 | 44 | 73 | 1.16 (0.81-1.66) |
| Dexamphetamine | 415 | 537 | 699 | 1.10 (0.94-1.29) | 328 | 370 | 332 | 1.11 (0.94-1.30) |
| Methylphenidate | 4819 | 4602 | 7432 | 1.06 (1.01-1.12) | 7725 | 4215 | 7215 | 1.01 (0.97-1.06) |
| Modafinil | 230 | 317 | 487 | 0.92 (0.75-1.12) | 393 | 347 | 534 | 0.91 (0.79-1.05) |
| Atomoxetine | 393 | 1274 | 700 | 1.02 (0.89-1.16) | 1551 | 1521 | 1513 | 0.96 (0.89-1.04) |
| Lisdexamphetamine | 1393 | 2604 | 2710 | 0.89 (0.82-0.96) | 1941 | 2122 | 2064 | 0.87 (0.82-0.94) |
| ADHD polytherapy | 558 | 1671 | 841 | 1.04 (0.92-1.17) | 924 | 1567 | 833 | 1.00 (0.91-1.09) |

ADHD, attention-deficit/hyperactivity disorder; aHR, adjusted hazard ratio; CI, confidence interval; SSDs, schizophrenia spectrum disorders; aHR = hazard ratios adjusted for age, sex, number of previous hospitalizations for psychosis, diagnosis of ADHD, substance use disorder, previous suicide attempts, previous use of clozapine, time-varying use of antipsychotics, antidepressants, mood stabilizers, drugs for addictive disorders, benzodiazepines and related drugs, and temporal order of ADHD drugs used in between-individual analyses.

*Comparison group = periods of non-use of both ADHD medication and antipsychotic treatment.

# Supplementary Table 2. *Most common causes of somatic hospitalization at 3 characters level among persons with SSD during periods of ADHD medication use.*

|  | **Events (N)** |
| --- | --- |
| L02 – Cutaneous abscess, furuncle and carbuncle | 347 |
| G40 – Epilepsy and recurrent seizures | 320 |
| J18 – Pneumonia, unspecified organism | 305 |
| M54 – Dorsalgia | 293 |
| G47 – Sleep disorders | 259 |
| K80 – Cholelithiasis | 244 |
| E11 – Type 2 diabetes mellitus | 226 |
| J15 – Bacterial pneumonia | 216 |
| E10 – Type 1 diabetes mellitus | 205 |
| K56 – Paralytic ileus and intestinal obstruction without hernia | 190 |
| K59 – Other functional intestinal disorders | 189 |
| J44 – Chronic obstructive pulmonary disease | 186 |
| I50 – Heart failure | 171 |

ADHD, attention-deficit/hyperactivity disorder; SSDs, schizophrenia spectrum disorders.

# Supplementary Figure 1. *Risk of hospitalization for neurological conditions* *associated with the use of ADHD medications among persons with SSDs (N=9,416).*


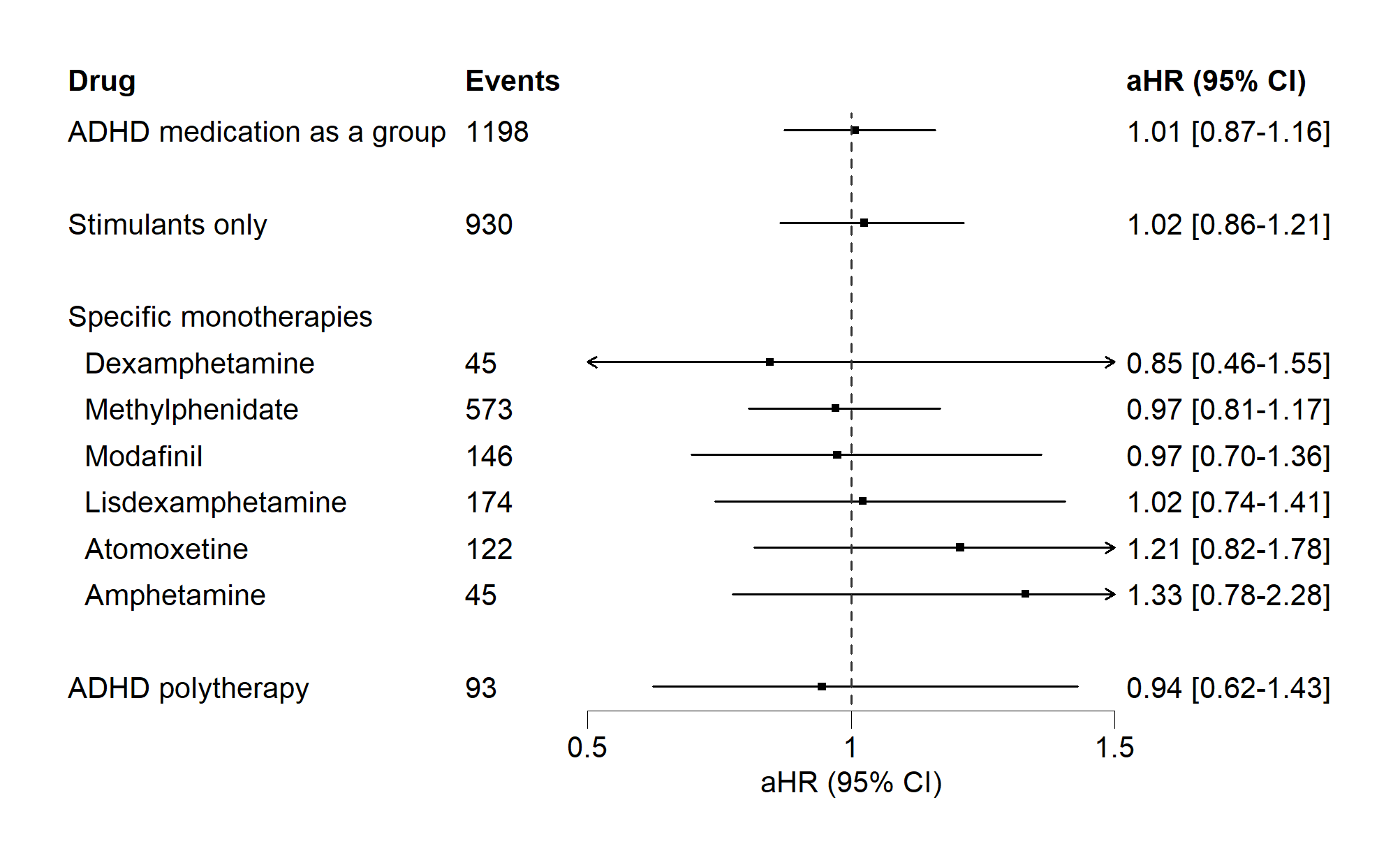


ADHD, attention-deficit/hyperactivity disorder; aHR, adjusted hazard ratio; CI, confidence interval; SSDs, schizophrenia spectrum disorders; aHR = hazard ratios adjusted for time-dependent covariates (i.e., time since cohort entry, temporal order of the ADHD medications used, and use of concomitant psychotropic drugs) in within-individual analyses.

# Supplementary Figure 2. *Risk of all-cause hospitalization/mortality associated with the use of specific ADHD medications among persons with SSD (N=9,416) using between-individual analyses.*


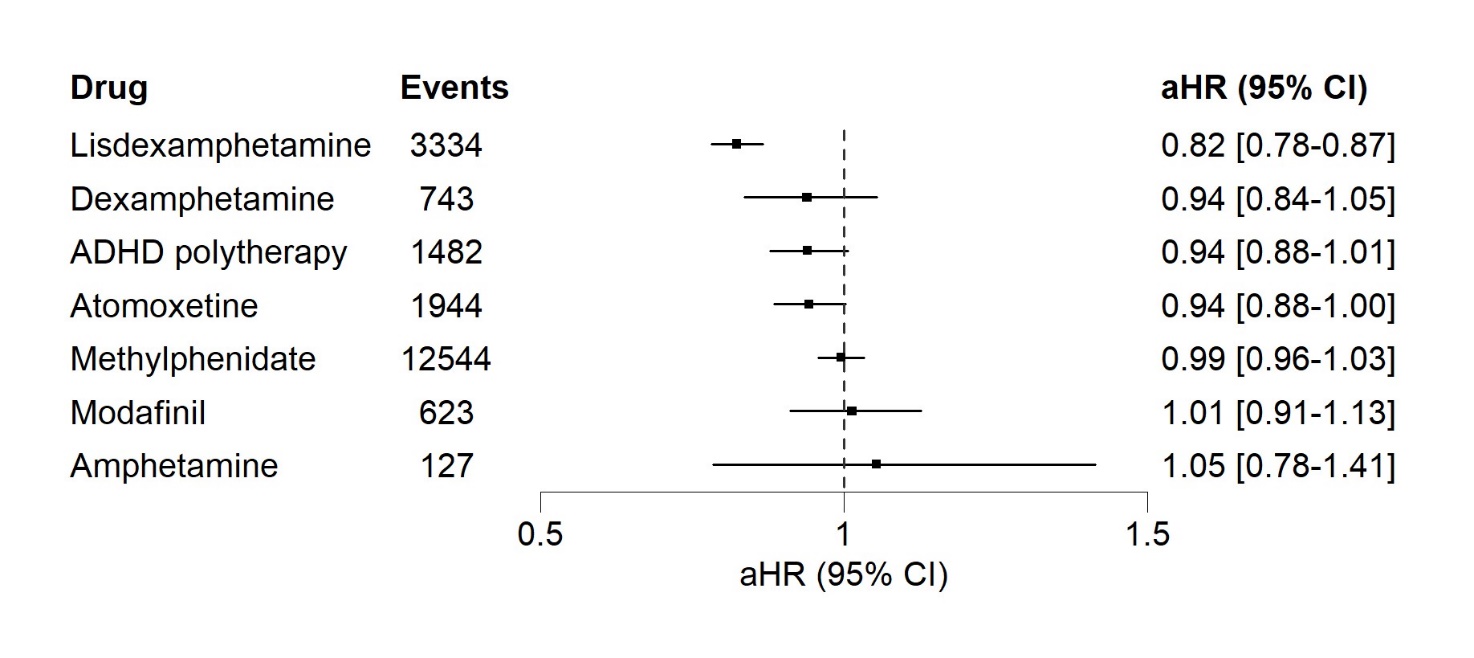


ADHD, attention-deficit/hyperactivity disorder; aHR, adjusted hazard ratio; CI, confidence interval; SSDs, schizophrenia spectrum disorders; aHR = hazard ratios adjusted for age, sex, disability pension, number of previous hospitalizations for psychosis, diagnosis of ADHD, substance use disorder, previous suicide attempts, previous use of clozapine, time-varying use of antipsychotics, antidepressants, mood stabilizers, drugs for addictive disorders, benzodiazepines and related drugs, and temporal order of ADHD drugs used in between-individual analyses.

# Supplementary Figure 3. *Risk of all-cause hospitalization/mortality associated with the use of specific ADHD medications among persons with SSDs (N=9,416), removing the first 30 days of ADHD medications non-use following their discontinuation.*


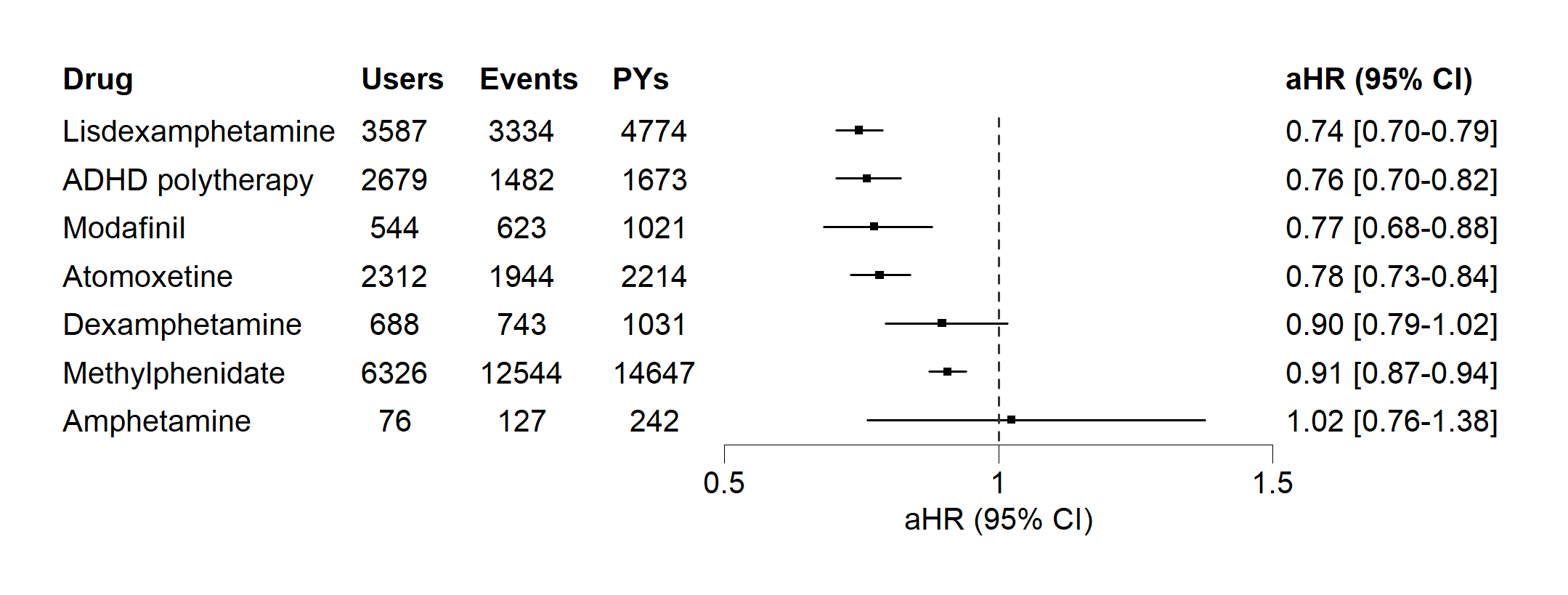


ADHD, attention-deficit/hyperactivity disorder; aHR, adjusted hazard ratio; CI, confidence interval; SSDs, schizophrenia spectrum disorders; PYs, person-years; aHR = hazard ratios adjusted for time-dependent covariates (i.e., time since cohort entry, temporal order of the ADHD medications used, and use of concomitant psychotropic drugs) in within-individual analyses.
